# No difference in complications between two-week vs. six-week duration of sling immobilization after reverse total shoulder arthroplasty

**DOI:** 10.1016/j.jseint.2023.07.015

**Published:** 2023-08-07

**Authors:** Matthew G. Alben, Neil Gambhir, Matthew T. Kingery, Robert Halpern, Aidan G. Papalia, Young W. Kwon, Joseph D. Zuckerman, Mandeep S. Virk

**Affiliations:** a Division of Shoulder and Elbow Surgery, Department of Orthopedic Surgery, NYU Grossman School of Medicine, NYU Langone Orthopedic Hospital, NYU Langone Health, New York, NY, USA; b Department of Orthopaedics and Sports Medicine, Jacobs School of Medicine and Biomedical Sciences, University at Buffalo, Buffalo, NY, USA

**Keywords:** Reverse, Total, Shoulder, Joint, Arthroplasty, Postoperative, Immobilization, Sling

## Abstract

**Background:**

The purpose of our study was to compare the outcomes and complications after a two- vs. six-week duration of sling immobilization following reverse total shoulder arthroplasty (rTSA).

**Methods:**

We conducted a retrospective review from our institutional database on 960 patients treated by primary rTSA between 2011 and 2021. Patients were separated into two cohorts of postoperative sling immobilization (a two-week and six-week group). Multivariate analysis was conducted to evaluate what factors were associated with patients experiencing either a postoperative complication or requiring reoperation.

**Results:**

A total of 276 patients were instructed to keep their operative arm in a sling for six weeks postoperatively, and 684 patients discontinued use at two weeks. There was no difference in postoperative complication rate (15.0% vs. 12.0%, *P* = .21), dislocation rate (*P* = .79), acromion stress fractures (*P* = .06), implant loosening (*P* = .15), and periprosthetic joint infections (*P* = .48) between the six- and two-week sling cohorts. In the immediate 90-day postoperative time period, no difference was seen in the reoperation rates (*P* = .73).

**Discussion:**

Shorter duration of sling immobilization (two weeks) does not incur additional risk of complications compared to standard duration (six weeks) of sling immobilization following rTSA.

Initially described for the treatment of rotator cuff tear arthropathy by Paul Grammont in 1987, reverse total shoulder arthroplasty (rTSA) procedures have seen over a 200% increase in the United States since its initial implant design approval by the Food and Drug Administration in 2004.^6^^,^^8^^,^^13^^,^^16^ As the number of rTSA increases across the United States and the world, variations in surgeon preferences have become more apparent with respect to indications and postoperative care. Systematic study of these variations in practice can help refine or improve patient care and determine or question clinical utility of certain practices.^12^^,^^18^

The duration of arm immobilization in a sling after rTSA is variable with no universal consensus on the ideal duration.^12^ There are a number of reasons that immobilization in sling is favored after rTSA. While doing so provides protection to the subscapularis tendon repair, the subscapularis tendon is not repaired or reparable in all rTSAs with prior studies having shown similar outcomes regardless of repair status.^7^^,^^9^^,^^15^^,^^19^ Some believe that immobilization in a sling allows formation of pseudocapsule around the shoulder, which provides internal soft tissue coverage especially in patients with cuff tear arthropathy.^3^^,^^11^^,^^14^ Furthermore, immobilization in a sling also protects against provocative motion (extension, adduction, and internal rotation) that can result in instability.^4^^,^^5^^,^^10^ However, the aforementioned reasons are not universally accepted.

There is considerable variation among surgeons with respect to duration of postoperative sling immobilization after rTSA with times ranging from less than a week to six weeks.^10^^,^^12^ The purpose of this retrospective study is to compare the one-year minimum postoperative outcomes and complications between short duration (two-weeks) vs. standard duration (six-weeks) immobilization after rTSA. We hypothesize that patients with a shorter duration of postoperative sling immobilization will have higher complication rate, including prosthetic instability and acromion stress fracture.

## Materials and methods

### Study design

A retrospective review of all patients undergoing primary rTSA between 2011 and 2021 was conducted from our institutional database. Study cohorts were divided into two groups based on the differing preference (two vs. six weeks) for sling immobilization duration by the fellowship-trained senior authors. The two-week sling immobilization cohort included patients from three surgeons, of which two surgeons have used a two-week period of sling immobilization throughout the study period. However, the third surgeon changed his preference to a shorter duration of immobilization (two weeks) after 2018. His preference for sling immobilization prior to the switch was six weeks. In order to avoid any crossover confounder, all of their cases six months prior and six months after the switch were not included in the study with the patients prior to switch included in the six-week cohort. The six-week sling immobilization cohort also included patients from one surgeon who has used a six-week period of sling immobilization following primary rTSA throughout the study period.

### Patient selection

Using Current Procedural Terminology code 23472, a total of 2788 patients were identified. Inclusion criteria required patients to have undergone rTSA with a minimum of one year follow-up. As this Current Procedural Terminology code captures patients who underwent aTSA, these patients as well as those requiring rTSA for revision surgery or proximal humerus fracture were excluded. Upon application of the aforementioned criteria, 960 patients were included in this study.

### Clinical sssessment and demographics

Chart review was conducted independently by two researchers to collect preoperative information including patient gender, race, body mass index (BMI), preoperative diagnosis, age at time of surgery, follow-up duration, prior shoulder surgery history, preoperative flexion and external rotation, and visual analog scale (VAS) pain scores. Intraoperative factors reviewed included additional procedures and concomitant shoulder pathology (rotator cuff or biceps tear), laterality, and inlay or onlay implant design. Though subscapularis repair is typically performed by each of the senior authors, the operative report was reviewed to determine if the tendon was amenable to repair intraoperatively. Postoperative complications, immobilization time, follow-up duration, postoperative flexion and external rotation, and VAS pain scores were recorded. Specific complications including rate of prosthetic dislocation (in first year vs. overall), acromion stress fracture, and risk of reoperation were compared between the two cohorts.

### Statistical analysis

Analysis was performed using R version 4.1.1 (The R Foundation for Statistical Computing, Vienna, Austria). The primary outcome of the study was reoperation following rTSA during the follow-up period. The secondary outcome was the presence of any complication following rTSA. Standard descriptive statistics were used to evaluate the demographics of the cohort. Chi-squared or Fisher’s exact test were used to compare categorical variables and t-tests were used to compare continuous variables between the two groups. Univariable and multivariable logistic regression analyses were conducted to determine which variables were associated with complications in patients with at least one year of follow-up after rTSA and to evaluate the effect of postoperative sling duration on subsequent complications. Similarly, multivariable logistic regression was used to evaluate the effect of sling duration on need for reoperation following rTSA. *P* values < 0.05 were considered to be statistically significant.

A priori sample size calculation was performed to determine the number of patients needed to detect a 5% increase in absolute risk associated with a shorter sling immobilization time compared to a six week duration. Based on an estimated 5% baseline risk of reoperation in the comparison group, α = 0.05, 1-β = 0.20, and an estimated 2:1 allocation ratio, a total sample size of 793 patients was required.

## Results

### Baseline demographic characteristics

A total of 960 patients underwent rTSA during the study period. Mean age of the cohort was 71.0 (± 9.1) years with a BMI of 30.0 (± 6.5 kg/m^2^) at the time of surgery. A total of 276 patients were instructed to keep their operative arm in a sling for six weeks postoperatively, and 684 patients discontinued use of the sling at two weeks postoperatively. The mean follow-up duration was 1.7 (± 1.8) years with no difference between the two groups (Table I). The two-week cohort had a higher number of patients with diagnosis of primary osteoarthritis (OA) (67% vs. 50%, *P* < .001) and received an onlay rTSA implant design (82% vs. 69%, *P* < .001) while the six-week cohort had a higher reported rate of subscapularis repair (96.4% vs. 69.1%, *P* < .001). No difference between the cohorts was found in respect to patient age (*P* = .24), gender (*P* = .19), BMI (*P* = .15), history of prior shoulder surgery (*P* = .85), or shoulder surgery laterality (*P* = .41).

**Table I:** Baseline characteristics for all patients undergoing reverse total shoulder arthroplasty.

	Time in sling		*P* value
	Six weeks, N = 276	Two weeks, N = 684	
Follow-up duration (years)	1.7 ± 1.8	1.6 ± 1.8	.86
Age (years)	70.5 ± 8.4	71.1 ± 9.3	.24
Sex			.19
Female	189 (68%)	436 (64%)	
Male	87 (32%)	248 (36%)	
BMI	30.5 ± 6.7	29.8 ± 6.4	.15
Diagnosis			**<.001**
Cuff arthropathy	135 (49%)	222 (32%)	
Instability	2 (0.7%)	4 (0.6%)	
Primary OA	139 (50%)	458 (67%)	
Prior shoulder surgery	66 (24%)	158 (23%)	.85
Laterality			.41
Left	110 (40%)	294 (43%)	
Right	166 (60%)	390 (57%)	
Implant design			**<.001**
Onlay	187 (69%)	562 (82%)	
Inlay	85 (31%)	122 (18%)	
Subscapularis tendon repair			**<.001**
Repaired	266 (96.4%)	473 (69.1%)	
Not repaired	4 (1.4%)	54 (7.9%)	
Unreported	6 (2.2%)	157 (23.0%)	
Complications			
Any complication	42 (15%)	82 (12%)	.21
Instability/dislocation	4 (1.4%)	14 (2.0%)	.79
Loosening	7 (2.5%)	8 (1.2%)	.15
Notching	1 (0.4%)	3 (0.4%)	1.00
PJI	8 (2.9%)	13 (1.9%)	.48
Acromion or scapular spine fracture	15 (5.4%)	20 (2.9%)	.06
Humeral shaft fracture	3 (1.1%)	5 (0.7%)	.70
Glenoid fracture	0 (0%)	3 (0.4%)	.56
Clavicle fracture	0 (0%)	2 (0.3%)	1.00
Coracoid fracture	0 (0%)	1 (0.1%)	1.00
Neurapraxia	6 (2.2%)	10 (1.5%)	.42
Other complication	3 (1.1%)	7 (1.0%)	1.00
Reoperation	22 (8.0%)	37 (5.4%)	.18
Reoperation within 90 days	2 (0.7%)	8 (1.2%)	.73

*BMI*, body mass index; *OA*, osteoarthritis; *PJI*, prosthetic joint infection.

Bolded values are those that meet statistical significance.

### Postoperative adverse events

In order to capture all complications that occurred during the study period, the entire cohort was first analyzed regardless of follow-up duration. Overall, 15% of the six-week sling group experienced a complication following rTSA compared to 12% of the two-week sling group (*P* = .21). There was no difference between the groups regarding the percentage of patients that required reoperation for any reason (8.0% vs. 5.4%, *P* = .18) or those undergoing reoperation within 90 days of the index procedure (0.7% vs. 1.2%, *P* = .73).

Further analyses were carried out using the subset of patients with at least one year of postoperative follow-up. Based on univariable logistic regression, patients with at least one year of follow-up who used a sling for only two weeks postoperatively had no difference in the odds of experiencing a complication compared to patients who used a sling for six weeks (odds ratio (OR) = 0.65, 95% confidence interval (CI) [0.40, 1.05], *P* = .08). Increased age at the time of rTSA (OR = 0.97, 95% CI [0.94, 0.99], *P* = .01) and primary OA as the indication for surgery (OR = 0.57, 95% CI [0.36, 0.91], *P* = .02) were associated with a lower odds of developing a complication. Gender, BMI, a history of prior shoulder surgery, and implant design were not associated with risk of complication (Table II).

**Table II:** Univariable and multivariable odds ratios (ORs) for experiencing a complication following reverse total shoulder arthroplasty for patients with at least 1 year of follow-up.

	No complication	Complication	OR (univariable)	OR (multivariable)
	N = 382 (80.6%)	N = 92 (19.4%)		
Time in sling				
Six weeks	105 (75.5%)	34 (24.5%)	-	-
Two weeks	277 (82.7%)	58 (17.3%)	0.65 (0.40-1.05, *P* = .08)	0.99 (0.56-1.80, *P* = .98)
Age				
Mean ± SD	71.3 ± 9.1	68.6 ± 8.5	**0.97 (0.94-0.99, *P* = .01)**	**0.97 (0.94-1.00, *P* = .04)**
Sex				
Female	255 (83.3%)	51 (16.7%)	-	-
Male	127 (75.6%)	41 (24.4%)	**1.61 (1.01-2.56, *P* = .04)**	1.40 (0.82-2.35, *P* = .21)
BMI				
Mean ± SD	29.3 ± 6.1	29.6 ± 7.3	1.01 (0.97-1.05, *P* = .71)	0.99 (0.95-1.03, *P* = .73)
Diagnosis				
Cuff arthropathy	128 (74.9%)	43 (25.1%)	-	-
Instability	4 (80.0%)	1 (20.0%)	0.74 (0.04-5.20, *P* = .79)	0.88 (0.04-7.83, *P* = .92)
Primary OA	250 (83.9%)	48 (16.1%)	**0.57 (0.36-0.91, *P* = .02)**	**0.54 (0.31-0.91, *P* = .02)**
Prior shoulder surgery				
No	285 (81.4%)	65 (18.6%)	-	-
Yes	97 (78.2%)	27 (21.8%)	1.22 (0.73-2.00, *P* = .44)	1.06 (0.59-1.84, *P* = .85)
Implant design				
Onlay	301 (81.1%)	70 (18.9%)	-	-
Inlay	78 (78.8%)	21 (21.2%)	1.16 (0.66-1.97, *P* = .60)	1.29 (0.67-2.42, *P* = .43)

*SD*, standard deviation; *BMI*, body mass index; *OA*, osteoarthritis.

Bolded values are those that meet statistical significance.

The same independent variables were then analyzed in a multivariable logistic regression to evaluate the effect of sling time on overall risk of complication following rTSA for patients with at least one year of follow-up. Increased age and a diagnosis of primary OA were again associated with lower odds of developing a complication. When controlling for the effect age, sex, BMI, indication for surgery, history of prior shoulder surgery, and implant design, there was no difference in overall odds of developing a complication after rTSA between patients who were in a sling for two weeks and those who were in a sling for six weeks (OR = 0.99, 95% CI [0.56, 1.80], *P* = .98).

**Figure 1:**
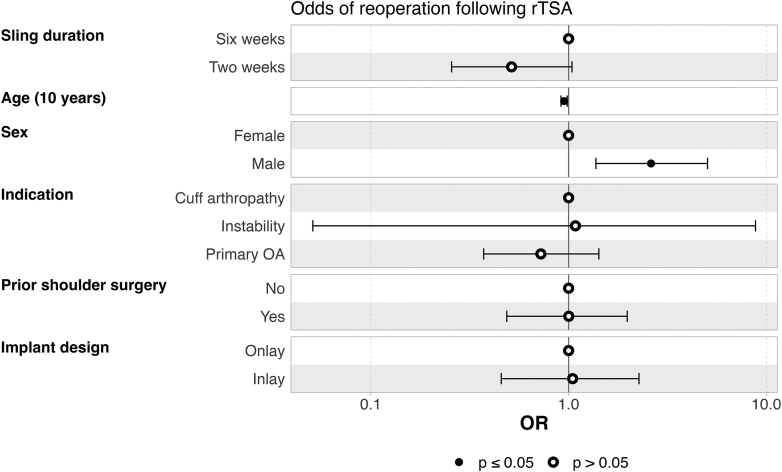
Odds ratios for reoperation following rTSA for patients with at least one year of follow-up postoperatively. *rTSA*, reverse total shoulder arthroplasty; *OA*, osteoarthritis; *CI*, confidence interval; *OR*, odds ratio.

To evaluate the effect of sling time on the risk of undergoing reoperation following rTSA, the subset of patients with at least one year of follow-up were included in a multivariable logistic regression analysis (Fig. 1). When controlling for age, sex, diagnosis, prior shoulder surgery, and implant design, discontinuing the sling after two weeks was associated with no difference in the odds of requiring reoperation for any reason compared to six weeks of sling time (OR = 0.51, 95% CI [0.26, 1.04], *P* = .06). Increased age at the time of surgery and female sex were associated with lower odds of reoperation.

### Clinical outcomes

To evaluate difference in postoperative shoulder stiffness after at least one year following rTSA, flexion and external rotation range of motion (ROM) at final follow-up were compared between groups. There was no difference in postoperative flexion (133.4 ± 30.3 degrees vs. 134.8 ± 32.1 degrees, *P* = .66) or change from preoperative flexion to postoperative flexion (42.7 ± 42.5 vs. 46.6 ± 44.9 degrees, *P* = .38) between the six-week sling group and the two-week sling group, respectively. Patients in the two-week sling group had a greater improvement in external rotation ROM after rTSA compared to the six-week sling group (18.2 ± 20.4 degrees vs. 10.4 ± 17.9 degrees, *P* < .001). However, the two-week sling group had worse preoperative external rotation compared to the six-week group (17.5 ± 17.9 degrees vs. 26.2 ± 15.3 degrees, *P* < .001), and there was no difference in absolute external rotation at final follow-up (35.7 ± 16.0 degrees vs. 36.5 ± 11.5 degrees, *P* = .56). Additionally, the two-week sling group had a lower VAS score of 1.76 when compared to 2.56 for the six-week sling group (*P* = .006).

## Discussion

Our study sought to compare the impact duration of sling immobilization had on one-year minimum outcomes and complications following rTSA with sling immobilization for the first six weeks postoperatively vs. those who discontinued sling use after two weeks. As such, we analyzed what factors influenced the odds of postoperative complications and reoperation, as well as the clinical outcomes in respect to ROM (flexion, external rotation) and VAS pain scores. No difference was found when comparing the two patient cohorts regarding the effect duration of postoperative immobilization had on postoperative complications (*P* = .98), reoperation rates (*P* = .06), and postoperative ROM (flexion, *P* = .66; external rotation, *P* = .56) between the two groups.

While this is the first study to specifically evaluate rTSA clinical outcomes after sling discontinuation at two vs. six weeks, postoperative rehabilitation has been of recent interest in the literature.^3^^,^^10^^,^^12^^,^^13^ A randomized control trial by Hagen et al compared the outcomes of rTSA patients who were immobilized for six weeks postoperatively (n = 54) vs. those who underwent immediate physical therapy with sling use (n = 53).^10^ No differences in American Shoulder and Elbow Score or ROM (flexion, abduction, external rotation) were reported at one year follow-up (*P* > .05). As for postoperative complications, the immediate-therapy group experienced isolated cases of revision surgery, an acromial stress fracture, and pulmonary embolism while the six-week group reported a postoperative dislocation, periprosthetic fracture, deep vein thromboembolism, and lymphedema. A systematic review by Bullock et al investigated postoperative protocols following rTSA.^3^ Specifically, six studies reported on sling use, with recommendations ranging from use as tolerated to six weeks, early postoperative deltoid isometric exercises, and ROM restrictions varying from no passive motion to precautionary range limits.

When comparing the duration of sling immobilization, our study found no difference in patient outcomes in respect to postoperative complications (*P* = .98) or those requiring reoperation for any reason (*P* = .06). Our hypothesis was that the patients with a shorter duration (2-weeks) of sling immobilization may be predisposed to higher risk of postoperative pain, instability, and acromion fractures. We acknowledge that these complications are multifactorial in etiology and sling immobilization alone is not the only cause for such outcomes or symptoms. If early mobilization and reducing the sling duration does not portend any obvious complication, there is not much advantage for a longer duration of sling immobilization (6-weeks) after surgery. Moreover, sling immobilization has its own disadvantages such as sleep disturbances and issues with activities of daily living, such as putting clothes on over or under the sling.

By specifically evaluating primary indications for elective rTSA, our study found OA (*P* = .02) and increased age (*P* = .04) to have a lower odds of postoperative complications. While there may be an inherent association between these findings as age is the greatest risk factor for OA, younger age (*P* = .006), rheumatoid arthritis (*P* = .01), and prior shoulder surgery (*P* < .001) have all been described to increase the risk of postoperative complications.^1^^,^^2^^,^^6^ In turn, underlying differences in the cohorts may affect the complication rates found within our patient population. Interestingly, our study found the two-week sling group to have a lower VAS score (1.76 vs. 2.56, *P* = .006) and greater improvement in external rotation (18.2 ± 20.4 degrees vs. 10.4 ± 17.9 degrees, *P* < .001). However, the previously reported minimally clinically important difference for VAS scores in rTSA patients is 1.4 and no difference was noted in the absolute final degree of external rotation (*P* = .56) between the two study cohorts.^17^ As the disparity in VAS scores between our groups is lower than the established minimally clinically important difference, it is likely that this finding is not clinically significant despite a statistically significant difference.

This study has several limitations. First, as this is an analysis of a large retrospective database, some patients were lost to follow-up and potential postoperative complications or revision surgeries were unable to be documented. Additionally, we are unable to verify if patients were compliant with sling immobilization as recommended in the immediate postoperative period. Second, due to limited patient reported outcome measurement data we were unable to evaluate the effect sling duration may have on patient perceived outcomes and satisfaction. Third, there was variability among three surgeons regarding their preference for subscapularis repair and clinical methodology of estimating internal rotation. Therefore, we did not compare the ROM or strength for internal rotation between the two cohorts, nor did we evaluate subscapularis repair healing using imaging. VAS pain scores and ROM were evaluated to provide a consistent metric to assess patient clinical improvement, though future studies are needed to further implore the effect sling immobilization has on a patient’s perception of improvement and postoperative outcomes. Lastly, potential factors that could have influenced outcomes may not have been assessed on multivariate analysis as they were unavailable in our institutional patient database. While future randomized control studies are needed to further assess patient outcomes at differing durations of postoperative immobilization, our study provides the foundation for direct comparison of two vs. six weeks immobilization outcomes after rTSA.

## Conclusion

Shorter duration of sling immobilization (two weeks) does not incur additional risk of complications compared to standard duration (six weeks) of sling immobilization following rTSA.

## Disclaimers

Funding: This study is funded by 10.13039/100006732NYU Langone Health, no external funding or support was received for completion of this study.

Conflicts of interest: Young W. Kwon: This author is a paid consultant for DJO Surgical.

Joseph D. Zuckerman: Apos Therapy, Inc, Stock; Hip Innovation Technology, Stock; Musculoskeletal Transplant Foundation, Board Member; SLACK Inc. Publishing, Royalties; Thieme Inc Publishing, Royalties; Wolters Kluwer Health Publishing, Royalties; Exactech Design Surgeon, Royalties.

Mandeep S. Virk: This author is a paid consultant for Exactech, Inc.

The other authors, their immediate families, and any research foundation with which they are affiliated have not received any financial payments or other benefits from any commercial entity related to the subject of this article.
